# A naturally-occurring 22-bp coding deletion in *Ugt86Dd* reduces nicotine resistance in *Drosophila melanogaster*

**DOI:** 10.1186/s13104-020-05035-z

**Published:** 2020-03-30

**Authors:** Stuart J. Macdonald, Chad A. Highfill

**Affiliations:** 1 Department of Molecular Biosciences, University of Kansas, 4043 Haworth Hall, 1200 Sunnyside Avenue, Lawrence, KS 66045 USA; 2 Center for Computational Biology, University of Kansas, Lawrence, KS 66047 USA

**Keywords:** *Ugt86Dd*, *Ugt35C1*, *Drosophila*, Nicotine, CRISPR, QTL, DSPR

## Abstract

**Objective:**

Segregating genetic variants contribute to the response to toxic, xenobiotic compounds, and identifying these causative sites can help describe the mechanisms underlying metabolism of toxic compounds. In previous work we implicated the detoxification gene *Ugt86Dd* in the genetic control of larval nicotine resistance in *Drosophila melanogaster*. Furthermore, we suggested that a naturally-occurring 22-bp deletion that leads to a stop codon in exon 2 of the gene markedly reduces resistance. Here we use homology directed CRISPR/Cas9 gene editing to specifically test this hypothesis.

**Results:**

We edited chromosome three from an inbred strain named A4 which carries the insertion allele at *Ugt86Dd*, successfully generated four alleles carrying the 22-bp *Ugt86Dd* deletion, and substituted edited chromosomes back into the A4 background. The original A4 strain, and an un-edited control strain in the same A4 background, show no significant difference in egg-to-adult or larva-to-adult viability on either control media or nicotine-supplemented media, and only slightly delayed development in nicotine media. However, strains carrying the 22-bp deletion showed reduced viability in nicotine conditions, and significantly longer development. Our data strongly suggest that the naturally-occurring 22-bp insertion/deletion event in *Ugt86Dd* directly impacts variation in nicotine resistance in *D. melanogaster*.

## Introduction

A principal aim of quantitative genetics is to describe the precise set of causative allelic changes that impact phenotype. While Quantitative Trait Locus (QTL) mapping studies have considerable power to identify loci impacting phenotype, alone their resolution does not typically allow for the specific causative genes/variants to be determined [1]. In many systems, CRISPR/Cas9 editing now allows the phenotypic effects of allelic substitutions to be precisely characterized in vivo [2]. This allows characterization of alternative alleles in an otherwise identical genetic background, making it possible to validate candidate variants identified by QTL mapping.

The *Drosophila* Synthetic Population Resource (DSPR) is a collection of Recombinant Inbred Lines (RILs) derived from a pair of highly-recombinant, synthetic populations initiated with a series of inbred founding *D. melanogaster* strains [3]. Previously we used the DSPR to identify a very large effect QTL contributing to larval nicotine resistance [4]. This QTL implicated a cluster of 10 UDP-glucuronosyltransferase detoxification enzyme-encoding genes including *Ugt86Dd*. This gene has recently been renamed to *Ugt35C1* (annotation symbol CG6633, FlyBase ID FBgn0040256 [5]), but we retain the original name for consistency with our previous work [4, 6].

Highfill et al. [6] supported the involvement of *Ugt86Dd* in nicotine resistance via RNAi and CRISPR/Cas9-induced mutagenesis; Both ubiquitous knockdown of gene expression, and the introduction of premature stop codons to the gene, resulted in reduced larval nicotine resistance. Highfill et al. [6] additionally hypothesized that the causative variant was a 22-bp InDel (insertion/deletion) event occurring in exon 2 of *Ugt86Dd*. The deletion allele induces a premature stop codon, and segregates in lines founding the DSPR and in other *D. melanogaster* populations [6]. This hypothesis was based on the following results [4, 6]. First, the inbred founder A4 is relatively resistant to nicotine, has the insertion allele, and shows significantly higher *Ugt86Dd* expression than A3, a relatively susceptible founder containing the deletion allele. Second, using a common genetic background, overexpression of the A4-derived insertion allele leads to significantly greater nicotine resistance than overexpression of the A3-derived deletion allele. Third, RILs that carry deletion-containing haplotypes at *Ugt86Dd* have much lower resistance on average. Finally, statistically accounting for the InDel status of each RIL during QTL mapping in the DSPR radically reduces the estimated phenotypic effect of the mapped locus, suggesting that the InDel—or a variant in linkage disequilibrium with it—is causative.

Here, to establish that the naturally-occurring 22-bp deletion decreases resistance to nicotine, we use CRISPR/Cas9 genome editing and homology directed repair to specifically delete the relevant 22-bp from the *Ugt86Dd* allele carried by the A4 strain. We do find that this deletion leads to a significant reduction in nicotine resistance.

## Main text

### Materials and methods

#### Fly strains

We used the same CRISPR/Cas9 injection strain as Highfill et al. [6]. Briefly, chromosome 3 from DSPR founder strain A4 [3], which contains a *Ugt86Dd* transcript with no deletions, was substituted into Bloomington *Drosophila* Stock Center (BDSC) strain 55821 [y^1^ M{vas-Cas9.RFP-}ZH-2A w^1118^], yielding an injection strain with the genotype *vasa*-Cas9;; A4. Following injection (see below), edited alleles were balanced using BDSC strain 5704 (w^1^; Sb^1^/TM3, Gal4-Hsp70, UAS-GFP, y^+^ Ser^1^), and subsequently substituted into the A4 background via a series of standard fly crosses.

DSPR founder strains A3 and A4 [3, 7] were used as controls in nicotine resistance assays; A3 possesses a 22-bp deletion in *Ugt86Dd*, while A4 has the insertion allele. We also used the three CRISPR/Cas9-derived strains generated by Highfill et al. [6]: A4-*Ugt86Dd*^*Del1*^ and A4-*Ugt86Dd*^*Del11*^ carry 1- and 11-bp deletions, respectively, in *Ugt86Dd*, while A4-*Ugt86Dd*^*wt*^ is un-edited and retains the same *Ugt86Dd* allele as founder A4. All three of these mutations are in the same background as the alleles generated in the current work.

#### Guide RNA plasmid

The Cas9 target is within the 22-bp insertion sequence present in the A4 *Ugt86Dd* allele, such that if this region is deleted the PAM sequence is also eliminated. The guide RNA (gRNA) plasmid was the same one used in Highfill et al. [6]. Briefly, we annealed 5′-phosphorylated sense and antisense oligos (5′-CTTCGTCACTACGAAGTCATTGTGG-3′ and 5′-AAACCCACAATGACTTCGTAGTGAC-3′), and cloned into the pU6-BbsI-chiRNA plasmid (Addgene, Cambridge, MA; plasmid 45946) using the protocol from Gratz et al. [8].

#### Editing and mutant identification

Around 300 *vasa*-Cas9;; A4 embryos were injected (BestGene, Inc.) with a mixture of the gRNA plasmid at 250 ng/μl and a single-stranded DNA repair molecule at 100 ng/μl. The sequence of the repair molecule was 5′-CTTTGCATTCGGGAAATACTTTTTGTAAACATTTCTCATATGTGGCAGGTGAACGAATTGACTTCGTAGTGATTTTCCAAGCGCTCCAGAAACGTCATCCGATCGGTTCGAGGAGAGGTC-3′, which represents a sequence 60 nucleotides both up- and downstream of the desired deletion. Virgin G_0_ animals were individually crossed to BDSC 5704 animals, and a number of F_1_ balancer-containing animals were further crossed to BDSC 5704. After these F_1_ crosses yielded eggs, the F_1_ animals were subjected to single-fly DNA isolation (Puregene Cell and Tissue Kit, 158388; Qiagen). The DNA from each F_1_ animal was then used to PCR amplify the region around the gRNA target site (forward oligo = 5′-ACGCTTTTGCTCAGCATTTT-3′, reverse oligo = 5′-GGCTGGGGATACCATTTCTT-3′, cycling conditions = 95 °C 2 min, 35 cycles of 95 °C 20 s/57 °C 25 s/72 °C 30 s, 72 °C 2 min). Subsequently PCR reactions were enzymatically-treated (5 units of exonuclease I, 2 units of shrimp alkaline phosphatase, 37 °C 1 h, 80 °C 15 min), and sent for Sanger sequencing using the forward PCR oligo (ACGT, Inc.). For four F_1_ edited animals (each derived from a different G_0_ animal) that contained the desired 22-bp deletion, we collected balanced F_2_ animals to establish stocks, and subsequently removed the balancers and substituted the edited third chromosomes back into the A4 background. The four resulting strains—A4-*Ugt86Dd*^*Del22*−*13H*^, A4-*Ugt86Dd*^*Del22*−*17B*^, A4-*Ugt86Dd*^*Del22*−*23C*^, and A4-*Ugt86Dd*^*Del22*−*26I*^—have the A4 background aside from the 22-bp deletion in *Ugt86Dd*.

#### First instar larva nicotine resistance assay

We employed the same larval nicotine resistance assay described in Marriage et al. [4] and Highfill et al. [6]. Briefly, all nine strains were expanded into multiple vials, and allowed to lay eggs on media supplemented with active yeast paste to elicit egg laying. For each strain, groups of 30 first instar larvae were manually collected and placed into 8 vials containing nicotine-supplemented media (0.18 μl/ml nicotine; N3876, Sigma) and into 4 vials containing, control, no-drug media. The phenotype for each replicate vial is the fraction of larvae that ultimately emerge as adults (counted 14 days after first instar larval collection). Raw data from this larval experiment is available in Additional file 1.

#### Embryo nicotine resistance assay

In an independent experiment we again expanded the nine strains to multiple vials, and allowed them to lay eggs. We then moved groups of 30 eggs directly to each of 10 vials containing either nicotine-supplemented or control media. Subsequently we counted the number of adults that had emerged 9, 10, 11, 12, 13, and 15 days following egg collection. Raw data from this embryo experiment is available in Additional file 2.

#### General phenotyping conditions

We used a cornmeal-yeast-molasses medium, and standard narrow *Drosophila* polystyrene vials. All animals were reared, crossed and assayed at 25 °C and 50% relative humidity, using a 12-h:12-h light:dark cycle.

#### Statistical analysis

All analysis was executed using base functions available in R [9]. See Additional file 3 for scripts.

### Results and conclusions

#### Development of edited strains containing a natural 22-bp deletion at *Ugt86Dd*

**Fig. 1:**
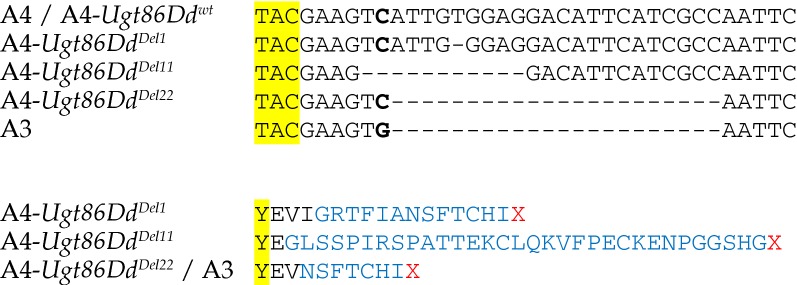
Sequences of tested *Ugt86Dd* alleles. The top panel shows a fraction of exon 2 from the gene. The leftmost “T” (rightmost “C”) corresponds to position 3R: 11127665 (3R: 11127626) in Release 6 of the *D. melanogaster* reference genome. The bottom panel shows the predicted polypeptide sequences resulting from strains containing deletions. The “TAC” codon results in the “Y” amino acid (both highlighted in yellow). A3 and A4 are inbred, but wild-derived, strains [3]. A4-*Ugt86Dd*^*wt*^, A4-*Ugt86Dd*^*Del1*^, and A4-*Ugt86Dd*^*Del11*^ were generated by Highfill et al. [6]. Four versions of A4-*Ugt86Dd*^*Del22*^ (*13H*, *17B*, *23C*, and *26I*) are identical for this region of the genome, and are new to the present study

Four of the founders of the DSPR, including A3, harbor a 22-bp coding deletion in *Ugt86Dd* that leads to a premature stop codon, while the 11 remaining founders, including A4, lack this deletion. Previous CRISPR editing generated strains carrying 1- and 11-bp deletions in *Ugt86Dd* in the A4 background [6], and here we generated four edited strains that carry the A3-like 22-bp deletion allele in the A4 background (Fig. 1). Since the edited chromosomes derive from different injected embryos, these four 22-bp deletion alleles are independent. All of the deletions result in premature stop codons, although differ in the predicted truncated polypeptides (Fig. 1). Note that the substitution adjacent to the 22-bp InDel that also distinguishes A3 from A4 (see the bolded base in Fig. 1) was left unchanged during the editing process.

#### A 22-bp deletion in *Ugt86Dd* leads to reduced larval nicotine resistance

**Fig. 2:**
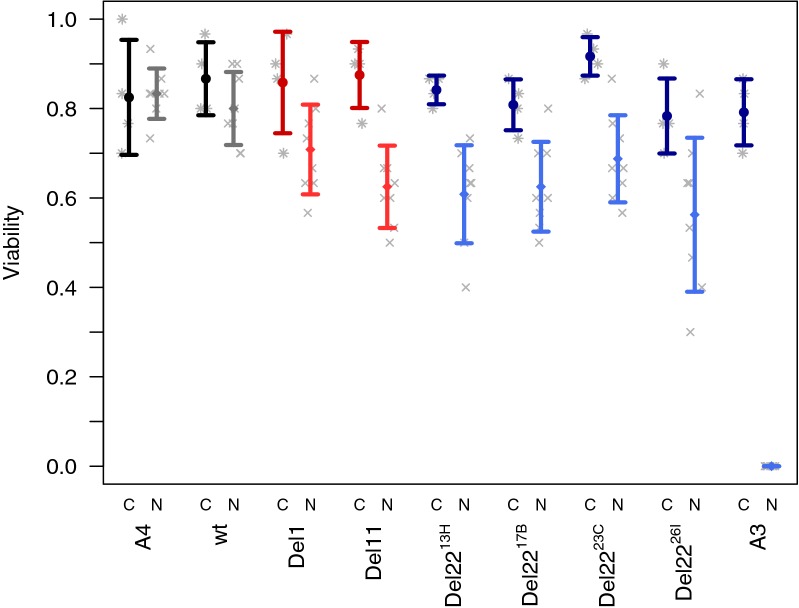
Larval resistance to nicotine is impacted by deletions in *Ugt86Dd*. Each strain was tested in both control (C) and nicotine (N) treatments. The fraction of adults emerging from each replicate vial is shown by asterisk and cross symbols, while the genotype/treatment means (± 1-SD) are shown by colored whiskers. Nicotine viability is lower than control viability for all strains with a deletion in *Ugt86Dd* (red and blue whiskers; see Table 1)

Founder strains A3 and A4, all 6 CRISPR/Cas9-edited strains, and one un-edited strain that was otherwise passed through the same injection/crossing scheme, were subjected to a larval nicotine resistance assay. Results are presented in Table 1 and Fig. 2. Neither founder A4 or the un-edited A4-*Ugt86Dd*^*wt*^ strain—which in principal should have identical genomes—show a significant difference in adult emergence between control and nicotine treatments. In contrast, founder A3 shows a radical difference between control and nicotine treatments; zero adults emerge from the nicotine treatment. As demonstrated previously by Highfill et al. [6] strains carrying CRISPR/Cas9-induced 1- and 11-bp deletions in *Ugt86Dd* show reduced adult emergence in the presence of nicotine, although the differences between treatments that we see here are lower than those observed by Highfill et al. [6], and the difference for A4-*Ugt86Dd*^*Del1*^ does not survive a nominal 10% statistical threshold. All four of the newly-generated strains that carry the A3-like 22-bp deletion engineered into the A4 strain background show significantly reduced adult emergence under nicotine conditions, and have similar phenotypes to those conferred by the 1- and 11-bp deletions (Table 1, Fig. 2). This result strongly supports the contention that the naturally-occurring 22-bp InDel event in *Ugt86Dd* is responsible for much of the QTL effect observed in Marriage et al. [4].

**Table 1:** Summary of nicotine resistance phenotypes

Genotype	InDel status	Larval assay		Embryo assay			
		Diff (N − C)^a^	*p*-value^b^	C^c^	N^c^	*p*-value^b^	Dev. delay (d)^d^
A4	Ins	0.01	0.908	0.76	0.72	0.159	0.24
A4-*Ugt86Dd*^*wt*^	Ins	− 0.07	0.230	0.77	0.83	0.100	0.26
A4-*Ugt86Dd*^*Del1*^	1-bp Del	− 0.15	0.071	0.67	0.53	0.007	1.26
A4-*Ugt86Dd*^*Del11*^	11-bp Del	− 0.25	0.001*	0.62	0.58	0.467	0.97
A4-*Ugt86Dd*^*Del22*−*13H*^	22-bp Del	− 0.23	< 0.001*	0.69	0.53	< 0.001*	1.24
A4-*Ugt86Dd*^*Del22*−*17B*^	22-bp Del	− 0.18	0.003*	0.61	0.44	0.009	1.41
A4-*Ugt86Dd*^*Del22*−*23C*^	22-bp Del	− 0.23	< 0.001*	0.56	0.45	0.092	1.49
A4-*Ugt86Dd*^*Del22*−*26I*^	22-bp Del	− 0.22	0.014	0.61	0.47	0.002*	1.62
A3	22-bp Del	− 0.79	< 0.001*	0.39	0.02	< 0.001*	3.83

^a^The difference between the fraction of adults emerging from nicotine (N) vials minus the fraction emerging from control (C) vials. Negative values imply that adult emergence is lower in the presence of nicotine. Also see Fig. 2

^b^The result of genotype-by-genotype Welch’s two sample *t*-tests comparing the per-vial fraction of adults emerging in nicotine and control treatments. Those *p*-values marked with an * are significant following a per-assay Bonferroni correction for multiple tests (i.e., 0.05/9)

^c^The fraction of adults emerging from embryos in control (C) vials or nicotine (N) vials

^d^The difference (in days, d) between the average emergence day of adults on nicotine media and that on control media. All values are positive, implying that development time is longer under nicotine conditions. All genotypes showed a significant developmental delay on nicotine media (Welch’s *t*-tests, *p* < 0.001)

Notably however, all engineered deletion-carrying strains remain considerably more resistant than founder A3, implying this line carries additional alleles that reduce its ability to counteract the toxic effects of nicotine. Indeed, using high-quality genomes of the DSPR founders Chakraborty et al. [7] report that a number of structural and copy number variants distinguish strains A3 and A4.

#### Deletions in *Ugt86Dd* lead to delayed development under nicotine conditions

We executed a similar experiment to that described above, but instead seeded vials with embryos rather than first instar larvae. As expected, egg-adult viabilities were generally lower than larvae-adult viabilities under both treatments (Table 1). However, the difference between these viability metrics among strains was marked; For instance, under control conditions A4 showed larvae-adult viability of 82.5% and egg-adult viability of 75.7%, while A3 showed viabilities of 79.2% and 39.3%, respectively. Nonetheless, as for the experiment initiated with first instar larvae, we found that deletions in *Ugt86Dd* typically led to a significant reduction in viability under nicotine conditions (Table 1). There was one notable exception to this pattern; A4-*Ugt86Dd*^*Del11*^ only showed a significant reduction in viability on nicotine media when using first instar larvae, and not when using embryos.

For this experiment we counted the number of adults emerging from each vial over several days. We found that nicotine routinely and significantly delayed adult emergence in all genotypes. However, compared to A4 and the un-edited A4-*Ugt86Dd*^*wt*^ strain, strains with CRISPR/Cas9 edits engineered into the A4 background exhibited 3.7–6.7 fold greater developmental delays (Table 1). Collectively, our experiments suggest that an intact *Ugt86Dd* gene limits nicotine toxicity in *D. melanogaster*, and the 22-bp deletion in the gene both decreases viability, and increases development time on media containing the drug.

## Limitations

Our work strongly suggests removal of a specific 22-bp of coding sequence from *Ugt86Dd* reduces nicotine resistance. However, we have not conducted the reverse experiment to determine whether “repairing” an allele that naturally contains the deletion (e.g., adding the 22-bp insertion sequence to strain A3) increases nicotine resistance as we would expect.

All of our work is conducted using strains endogenously expressing Cas9 via integrated transgenes, entailing multiple crosses with balancer chromosomes to substitute edited chromosomes into an appropriate background. Since gene conversion can move genetic information from the balancer to the non-balancer homolog [10], there is the potential for our CRISPR/Cas9-derived strains to segregate for variants outside of the region of *Ugt86Dd* we have sequenced. Moreover, Cas9 can lead to off-target mutations during the editing process [11]. Such variants could impact the interpretation of our data, but we have not conducted whole genome sequencing of our strains to uncover them.

## Supplementary information

**Additional file 1.** Raw data from the first instar larva nicotine resistance assay. The “strain_id” column gives the name of the strain as used in the main text, the “brief_lab_id” column gives a Macdonald lab identifier of each strain, and ”genotype_info” gives information about the allele present at the *Ugt86Dd* gene in the strain. The “media_treatment” column states whether the strain was assayed on control, no-nicotine media, or on nicotine-supplemented media. The “replicate_vial” column gives a number encoding each within-strain/within-treatment replicate assay vial, “number_first_instar_larvae” defines the number of larvae used to seed each vial, while “number_emerged_adults” is the number of adults emerging from the vial at day 14 following larval collection.

**Additional file 2.** Raw data from the embryo nicotine resistance assay. The “strain_id” column gives the name of the strain as used in the main text, and the “brief_lab_id” column gives a Macdonald lab identifier of each strain. The “replicate_vial” column gives a number encoding each within-strain/within-treatment replicate assay vial. The “media_treatment” column states whether the strain was assayed on control, no-nicotine media, or on nicotine-supplemented media. The “number_eggs” column defines the number of eggs used to seed each vial. Finally, the series of columns beginning with “number_emerged_adults_day” defines the number of adults that emerged on a particular day (9, 10, 11, 12, 13, 15) following egg collection.

**Additional file 3.** Contains R scripts allowing the results, including Fig. 2, from the manuscript to be recapitulated.

## Data Availability

All data generated in this study are provided in Additional files 1 and 2. R scripts to recapitulate analyses presented here are in Additional file 3. Fly strains generated are available on request to the corresponding author.

## Abbreviations

QTL: Quantitative Trait Locus
CRISPR: Clustered Regularly Interspaced Short Palindromic Repeats
DSPR: *Drosophila* Synthetic Population Resource
RIL: Recombinant Inbred Line
InDel: Insertion/deletion
BDSC: Bloomington *Drosophila* Stock Center
gRNA: Guide RNA
DNA: Deoxyribonucleic acid
PCR: Polymerase Chain Reaction
